# N-terminal pro B-type natriuretic peptide (NT-proBNP): a possible surrogate of biological age in the elderly people

**DOI:** 10.1007/s11357-020-00249-2

**Published:** 2020-08-11

**Authors:** Antonio Muscari, Giampaolo Bianchi, Paola Forti, Donatella Magalotti, Paolo Pandolfi, Marco Zoli, The Pianoro Study Group

**Affiliations:** 1 Department of Medical and Surgical Sciences, University of Bologna-S.Orsola-Malpighi Hospital, Via Albertoni, 15, 40138 Bologna, Italy; 2 Medical Department of Continuity of Care and Disability, S.Orsola-Malpighi Hospital, Bologna, Italy; 3 Epidemiological and Health Promotion Unit, Department of Public Health, AUSL Bologna, Bologna, Italy

**Keywords:** NT-proBNP, Mortality, Biological age, Epidemiological studies

## Abstract

NT-proB-type natriuretic peptide (NT-proBNP) increases with age and is associated with all-cause mortality. With this study, we assessed its possible utilization as a marker of biological age in comparison with other variables. The study included 1079 non-institutionalized elderly subjects (mean age 72.8 ± 5.5 years, 561 women). Baseline measurements were performed of serum NT-proBNP and of some laboratory variables previously utilized to estimate biological age (creatinine, albumin, C-reactive protein, cholesterol, blood glucose, leukocytes, lymphocytes, hemoglobin, mean cell volume). During 7 years of follow-up, 114 all-cause deaths occurred. The logarithm of NT-proBNP was the most age-related parameter (*r* = 0.35, *P* < 0.0001). Its relationship with mortality, according to Cox regression and ROC curve (AUC = 0.707, 95% CI 0.654–0.759), was stronger than that of all other variables, including age. In multivariate analysis, only NT-proBNP and age remained independently associated with mortality. The regression lines between age and NT-proBNP (pg/ml) allowed a separate estimation of biological age (“proBNPage”) for men (= [log(NT-proBNP) + 1.2068]/0.0827) and for women (= [log(NT-proBNP) − 1.5258]/0.0478). The hazard ratio of all-cause mortality for the fifth quintile of proBNP age (≥ 85 years) compared with the first quintile (< 61 years) was 7.9 (95% CI 3.6–17.5). Similarly, the difference between pro-BNPage and chronological age was associated with a hazard ratio of 3.5 in the fifth quintile (95% CI 1.9–6.4) and was associated with disease count (*P* for trend = 0.0002). In conclusion, NT-proBNP was the best indicator of biological age, which can be estimated by simple formulas and might be used for prognostic purposes or as a surrogate end point in epidemiological and intervention studies.

## Introduction

The difference between biological and chronological age is easily understandable to everybody, as we all know persons who show more or fewer years than their age and who behave accordingly. Biological age may be defined as a measure, more accurate than chronological age, of how really close we are to death. Finding an enough precise method to determine biological age is more difficult. However, this finding could lead to two important applications: (1) a reliable prognostic index and (2) a surrogate end-point to be used in the search for anti-aging treatments in the place of mortality, which can only be determined with long longitudinal studies.

Keeping in mind these premises, a few characteristics have been defined for the ideal indicator of biological age (Jylhävä et al. 2017; Johnson 2006): (1) it must be correlated with chronological age; (2) it must be associated with mortality, possibly better than chronological age; (3) it must be associated with morbidity; (4) it should be associated with the aging process, rather than with the effects of diseases; (5) it should be easily and repeatedly measurable; (6) it should have a chance to change due to therapeutic interventions.

The studies on this subject have been numerous, suggesting several potential indicators of biological age. These may be grouped into 6 main categories:

1. Measures of DNA methylation (epigenetic clock) (Hannum et al. 2013; Horvath 2013), the indicators most correlated with chronological age, probably because DNA methylation is a time-dependent process. They have however a mild-to-moderate association with mortality, with better results with cancer than with cardiovascular mortality (Chen et al. 2016; Perna et al. 2016)
2. Telomere length in leukocytes (Sanders and Newman 2013), perhaps the most popular and reliable indicator of biological age, although not routinely measurable
3. Proteomic and metabolomic indices (Menni et al. 2015; Johnson et al. 2019), which however are supported by still scarce and poorly conclusive studies
4. Magnetic resonance imaging to assess brain age, which is associated with cognitive decline and biological age (Cole et al. 2018; Elliott et al. 2019)
5. Composite frailty or deficit indices based on the combination of many clinical-anamnestic-functional variables (Mitnitski et al. 2001; Abete et al. 2017; Rockwood et al. 2017; Kim and Jazwinski 2015)
6. Composite indices mainly based on the combination of laboratory variables (Yoo et al. 2017; Belsky et al. 2017; Liu et al. 2018; Waziry et al. 2019). These are easily and repeatedly measurable, including routine variables such as albumin, creatinine, blood glucose, uric acid, C-reactive protein, and leukocytes, many of which, even when considered alone, are associated with mortality, morbidity, and chronological age.

As far as the latter category is concerned, rather surprisingly, some very promising biochemical parameters have not been considered yet. These are natriuretic peptides, particularly B-type natriuretic peptide (BNP) and the N-terminal fragment of its precursor (NT-proBNP). Both are mainly released by stressed cardiomyocytes in cardiac walls and are therefore important indicators of heart disease and cardiovascular mortality (McDonagh et al. 2004; Galvani et al. 2004; Ponikowski et al. 2016; Wang et al. 2004). However, they also predict mortality in subjects free of heart disease (McKie et al. 200; Wannamethee et al. 2011; Muscari et al. 2013), are associated with non-cardiovascular and all-cause mortality (Muscari et al. 203) and progressively increase with age (Redfield et al. 2002; Wang et al. 2002; Luchner et al. 2013). Within the Pianoro Study, an epidemiological investigation concerning the elderly population resident in three rural municipalities of Northern Italy, we have previously reported the association between baseline NT-proBN*P* values and mortality during a 7-year follow-up (Muscari et al. 2013). In the present study, we have assessed how NT-proBNP and some biochemical variables utilized in previous studies were associated with chronological age and all-cause mortality, with the goal of identifying, among them, the best indicator of biological age in the elderly population.

## Methods

### Subjects

The Pianoro Study has been described in previous publications (Muscari et al. 2007; 2013). Shortly, starting from November 2003, all of the 3255 inhabitants of the Pianoro municipality (Northern Italy) aged ≥ 65 years were invited to participate in the study. Two thousand twenty-two subjects returned a preliminary postal questionnaire and, of them, 1163 agreed to be subsequently examined in our laboratories. The characteristics of the subjects who returned their postal questionnaires, but did not participate in the second phase, have previously been reported (Muscari et al. 2007). Forty-eight subjects were excluded due to missing data and further 36 subjects with a history of heart failure or atrial fibrillation (the main conditions associated with high NT-proBNP levels) were also excluded. Finally, 1079 subjects aged 65–93 years at the time of enrolment (mean age ± SD 72.8 ± 5.5 years, 518 men and 561 women) remained available for our analyses.

This study was approved by our joint University-Hospital Ethical Committee, and all participants provided their signed informed consent.

### Baseline variables

The subjects with a history of diabetes and those with fasting blood glucose ≥ 126 mg/dl were considered diabetic. The subjects under lipid lowering treatment and/or with serum levels of total cholesterol ≥ 200 mg/dl were considered hypercholesterolemic. The subjects under anti-hypertensive treatment and those with systolic blood pressure ≥ 140 mmHg and/or diastolic blood pressure ≥ 90 mmHg were considered hypertensive.

Venous blood sampling was performed in the morning, after a 12-h fast. All measurements were performed in the same laboratory and on the same day of sampling, using commercially available kits. In particular, serum levels of NT-proBNP were measured by an electrochemiluminescence immunoassay (proBNP Elecsys, Roche Diagnostics, Mannheim, Germany). The linear range of detection of NT-proBNP was of 5–35,000 pg/ml. The coefficients of variation for intra- and inter-assay measurements of NT-proBNP were, respectively, 4% and 5% for “low” mean levels (210 pg/ml) and 6% and 7% for higher mean levels (4400 pg/ml). The serum levels of high-sensitivity C-reactive protein (CRP) were measured with an immunoturbidimetric method (Tina-quant CRP-Latex, Roche Diagnostics, Mannheim, Germany). The complete blood count was obtained by an automated counter (Bayer ADVIA 120), and erythrocyte sedimentation rate (ESR) was automatically measured by the stopped-flow technique in a capillary microphotometer (Alifax Test 1 System).

### Epidemiological and statistical analysis

Participant survival ranged between 22 and 2647 days, with 114 deaths during a median follow-up time of 2458 days (6.7 years), until March 10, 2011. Morbidity was assessed by direct interview of the subject, relatives or caregivers, with assessment also of the available documentation. For disease count, all health problems were recorded either within organ categories (for example, gastroenterological, respiratory, urinary) which were further subdivided into acute and chronic or as previous important diseases (such as myocardial infarction or stroke).

The continuous variables were described as mean ± standard deviation (SD), or as median and interquartile range, in relation to their distribution (respectively Gaussian or non-Gaussian). The comparisons of such variables between 2 groups were tested, respectively, by Student’s *t* test for unpaired data or Mann-Whitney’s *U* test, while the comparisons among 3 or more groups were assessed with one-way ANOVA and linearity test. The comparisons between percentages were assessed by *χ*^2^ test.

Regressions and correlations were assessed by linear regression. For this analysis, the continuous variables with skewed distribution (NT-proBNP, ESR, lymphocytes, leukocytes, creatinine, mean cell volume [MCV], blood glucose and CRP) were preliminarily normalized by natural logarithmic transformation.

The associations with all-cause mortality were assessed by Cox proportional hazards regressions, considering both one variable at a time and all variables together. In these analyses, the Wald statistics was used to compare the strength of association with mortality of the variables. Cox regressions were also used to assess the hazard ratios (HR) and 95% confidence intervals (CI) associated with variable quintiles, using the low quintile as a reference.

In addition, receiver operating characteristic (ROC) curves with their areas under the curves (AUC) and 95% CIs were generated to assess the ability of each variable to distinguish deceased subjects from survivors.

Analyses were performed using SPSS Statistics v. 22 (IBM, Armonk, New York, USA). Two-tailed tests were used throughout, and *P* values < 0.05 were considered significant.

The main demographic, clinical, and laboratory characteristics of our sample of elderly people are illustrated in Table 1, with reference to survivors, deceased, and all subjects.

**Table 1:** Characteristics of the study sample

Characteristic	All	Survivors	Deceased	*P* value
	(*N* = 1079)	(*N* = 965)	(*N* = 114)	
Age (years)	72.8 ± 5.5	72.3 ± 5.0	77.0 ± 7.0	< 0.0001
Female	561 (52.0)	512 (53.1)	49 (43.0)	0.04
BMI (kg/m^2^)	26.5 ± 4.0	26.5 ± 4.1	25.9 ± 4.0	0.11
Hypertension	924 (85.6)	828 (85.8)	96 (84.2)	0.65
Hypercholesterolemia	843 (78.1)	765 (79.3)	78 (68.4)	0.008
Diabetes	149 (13.8)	123 (12.7)	26 (22.8)	0.003
Ever smoker	472 (43.7)	404 (41.9)	68 (59.6)	0.0003
Previous myocardial infarction	58 (5.4)	48 (5.0)	10 (8.8)	0.09
Previous stroke	26 (2.4)	19 (2.0)	7 (6.1)	0.006
Disease count	1 [0–2]	1 [0–2]	1 [1–2]	0.001
Blood glucose (mg/dl)	97 [90–108]	97 [90–108]	97 [88–108]	0.56
Cholesterol (mg/dl)	217.2 ± 37.6	218.3 ± 37.3	207.7 ± 38.6	0.004
Albumin (g/dl)	4.30 ± 0.30	4.31 ± 0.30	4.23 ± 0.32	0.02
Hemoglobin (g/dl)	13.9 ± 1.3	14.0 ± 1.3	13.6 ± 1.4	0.001
MCV (fl)	89.2 [86.3–92.1]	89.2 [86.3–92.0]	89.1 [85.8–93.3]	0.71
Leukocytes (× 10^9^/l)	5.90 [4.96–6.89]	5.86 [4.92–6.82]	6.43 [5.39–7.46]	0.001
Lymphocytes (%)	29.1 [24.5–33.7]	29.4 [24.9–33.8]	27.5 [21.4–32.2]	0.003
Creatinine (mg/dl)	0.94 [0.81–1.08]	0.93 [0.81–1.07]	1.01 [0.87–1.18]	0.0003
ESR (mm/h)	17 [11–30]	17 [11–29]	23 [11–35]	0.01
CRP (mg/dl)	0.19 [0.10–0.39]	0.19 [0.09–0.38]	0.20[0.12–0.51]	0.12
NT-proBNP (pg/ml)	132 [75–244]	124 [72–224]	245 [148–506]	< 0.0001

Values are mean ± SD, or median [25th–75th percentile], or number (percentage)

*BMI* body mass index, *CRP* C-reactive protein, *ESR* erythrocyte sedimentation rate, *MCV* mean cell volume

Table 2 shows the linear regressions and the correlations of chronological age with the laboratory variables. Several variables were preliminarily log-transformed due to their asymmetric log-normal distribution. The variables are listed in decreasing order of Pearson’s *r* coefficient. The logarithm of NT-proBNP was the variable most correlated with age (*r* = 0.35, *P* < 0.0001).

**Table 2:** Linear regressions and correlations with chronological age

Dependent variable	Slope	Intercept	Pearson’s r	*P* value
Log (NT-proBNP)	0.0633	0.2983	0.350	< 0.0001
Hemoglobin	− 0.0542	17.8879	0.230	< 0.0001
Log (ESR)	0.0211	1.2860	0.149	< 0.0001
Log (Lymphocytes)	− 0.0063	3.8031	0.132	< 0.0001
Log (Creatinine)	0.0049	− 0.4120	0.123	< 0.0001
Albumin	− 0.0062	4.7332	0.081	0.008
Cholesterol	− 0.5099	254.4	0.074	0.01
Log (MCV)	0.0009	4.4173	0.073	0.02
Log (Blood Glucose)	− 0.0014	4.7131	0.042	0.16
Log (Leukocytes)	− 0.0014	1.8721	0.031	0.32
Log (CRP)	0.0011	− 1.6709	0.005	0.87

*CRP* C-reactive protein, *ESR* erythrocyte sedimentation rate, *MCV* mean cell volume

Table 3 shows the associations of the same variables, plus age, and disease count, with all-cause mortality. The associations were assessed by univariate Cox regression, multivariate Cox regression, and area under the ROC curve and are listed in decreasing order of the Wald statistics derived from univariate Cox regression. NT-proBNP was associated with the greatest Wald statistics (both in univariate and multivariate regression) and with the largest AUC. In multivariate Cox regression, NT-proBNP and chronological age were the only variables independently associated with mortality.

**Table 3:** Relationships with all-cause mortality

Independent variable	Univariate Cox regression		Multivariate Cox regression		ROC curve	
	Wald*	*P* value	Wald	*P* value	AUC	95% CI
NT-proBNP	109.4	< 0.0001	32.8	< 0.0001	0.707	0.654–0.759
Chronological age	70.5	< 0.0001	30.9	< 0.0001	0.695	0.638–0.752
Creatinine	20.7	< 0.0001	1.5	0.23	0.603	0.545–0.660
Disease count	17.5	0.0003	2.2	0.14	0.592	0.534–0.649
ESR	11.6	0.0007	1.2	0.28	0.572	0.513–0.631
Hemoglobin	− 9.8	0.002	0.001	0.98	0.589	0.533–0.646
Lymphocytes	− 8.5	0.004	0.2	0.64	0.584	0.526–0.643
Cholesterol	− 8.4	0.004	1.1	0.29	0.582	0.524–0.639
Leukocytes	7.8	0.005	3.8	0.051	0.592	0.535–0.649
CRP	6.1	0.01	0.1	0.71	0.547	0.487–0.607
Albumin	− 5.9	0.02	0.8	0.37	0.566	0.509–0.622
MCV	0.6	0.45	0.2	0.89	0.511	0.449–0.572
Blood glucose	0.2	0.65	0.1	0.81	0.483	0.422–0.545

*AUC* area under curve, *CRP* C-reactive protein, *ESR* erythrocyte sedimentation rate, *MCV* mean cell volume, *ROC* receiver operating characteristic

*Minus signs denote inverse relationships. The AUCs of the variables inversely associated with mortality have been symmetrically inverted to allow direct comparison with other AUCs

According to these assessments, we concluded that the best single indicator of biological age, among laboratory variables, was NT-proBNP. Table 4 confirms that also in our sample, as already known (Redfield et al. 2002; Wang et al. 2002; Luchner et al. 2013), NT-proBNP levels were on average higher in women than in men (although the table shows that the difference mainly concerned the “young old” group, namely, the subjects aged 65–74 years). Thus, NT-proBNP values had to be assessed separately in the two sexes.

**Table 4:** NT-pro BNP values according to age and sex

Age group	Men	Women	*P* value
All ages	119 [59–238] (*N* = 518)	143 [91–246] (*N* = 561)	0.0001
65–74 years	98 [52–179] (*N* = 379)	128 [82–214] (*N* = 383)	< 0.0001
75–84 years	201 [99–426] (*N* = 127)	198 [110–359] (*N* = 157)	0.89
≥ 85 years	361 [241–583] (*N* = 12)	277 [197–393] (*N* = 21)	0.41

Values, in picograms per milliliter, are medians [25th–75th percentile]

These are the regression lines of the logarithm of NT-proBNP with age, separately for men and women:

1 $$ \log \left(\mathrm{NT}-\mathrm{proBNP}\right)\ \mathrm{men}=0.0827\times \mathrm{Age}\hbox{--} 1.2068\ \left(r=0.384,P<0.0001\right) $$

2 $$ \log \left(\mathrm{NT}-\mathrm{proBNP}\right)\ \mathrm{women}=0.0478\times \mathrm{Age}+1.5258\ \left(r=0.319,P<0.0001\right) $$

The intersection point of the two lines can be calculated by solving the equation that is obtained by matching the terms on the right of Eqs. (1) and (2). The result is an age of 78.3 years, corresponding to the NT-proBNP level of 194 pg/ml for both sexes. Beyond that age, values of men become higher than those of women.

Inverting the two formulas, a possible estimate of biological age can be obtained, in years, starting from an NT-proBNP value. We will call this estimate “proBNP age”:

3 $$ \mathrm{proBNP}\ \mathrm{age}\ \mathrm{men}=\left[\log \left(\mathrm{NT}-\mathrm{proBNP}\right)+1.2068\right]/0.0827 $$

4 $$ \mathrm{proBNP}\ \mathrm{age}\ \mathrm{women}=\left[\log \left(\mathrm{NT}-\mathrm{proBNP}\right)-1.5258\right]/0.0478 $$

**Fig. 1:**
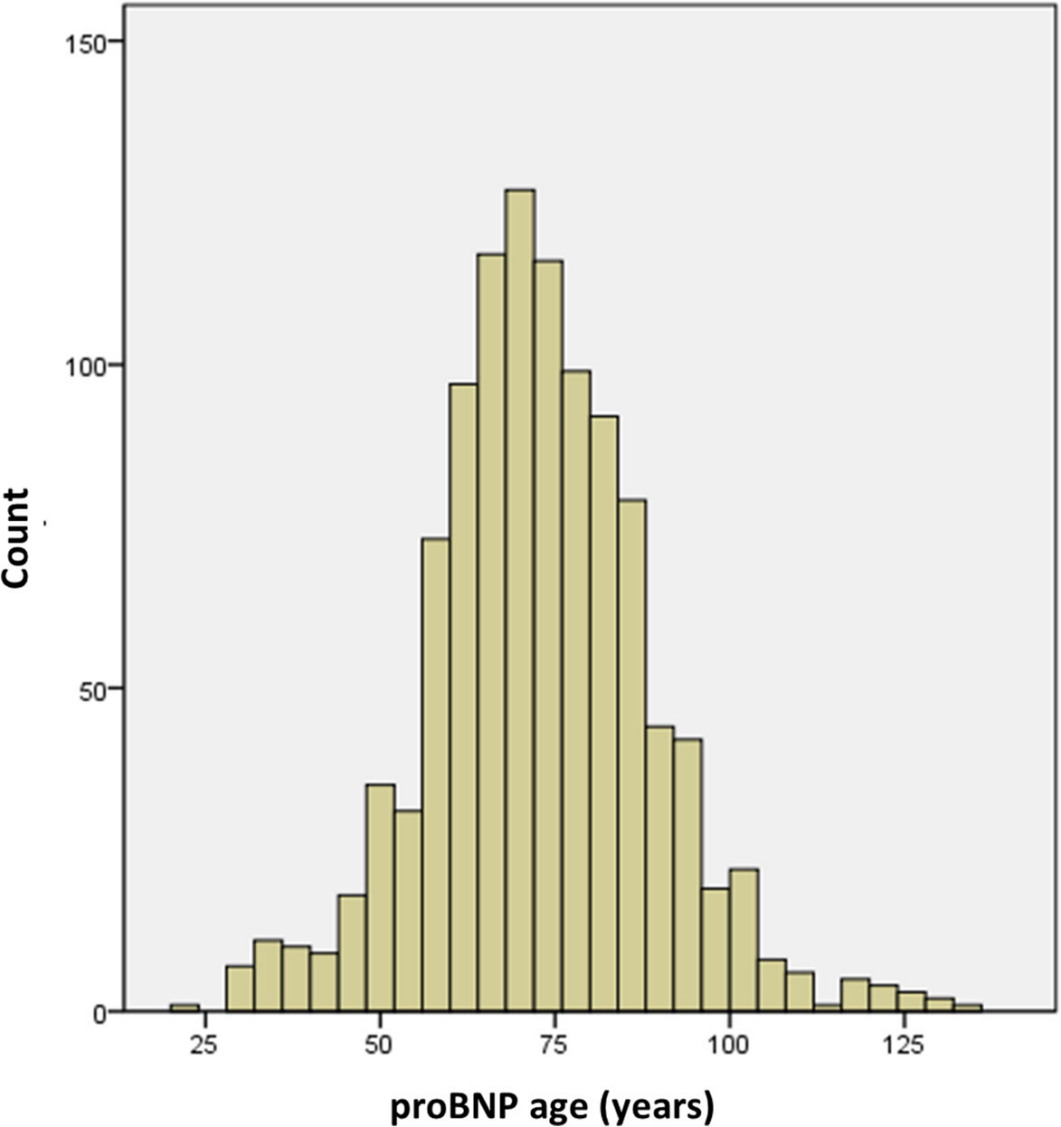
Histogram of proBNP age, showing an almost normal distribution

The values of proBNP age of the two sexes can now be treated together, and, in fact, Fig. 1 shows that pro-BNP age distribution is almost perfectly normal, with the same mean of chronological age (72.8 years) but with a larger S.D. (15.9 vs. 5.5 years).

**Fig. 2:**
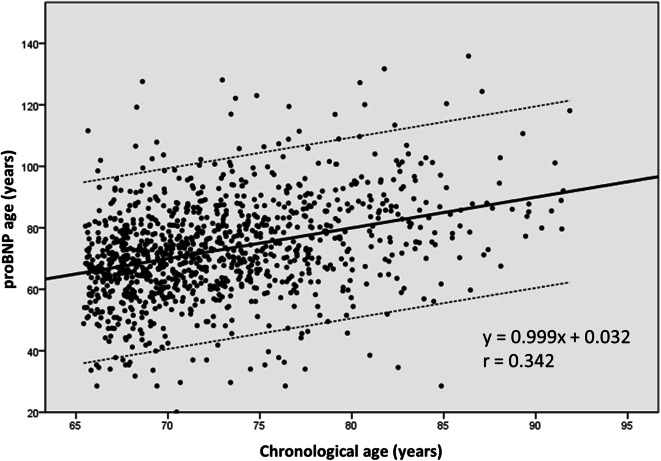
Regression line and 95% confidence interval between chronological age and proBNP age

Figure 2 shows the linear relationship between chronological and proBNP age. This is its equation:

5 $$ \mathrm{proBNP}\ \mathrm{age}=0.999\times \mathrm{Age}+0.032\ \left(r=0.342,P<0.0001\right) $$

Since the beta coefficient is very close to 1 and the constant is very close to 0, the values of proBNP age on the regression line almost coincide with the corresponding values of chronological age, but the variability of the other values is wide.

**Fig. 3:**
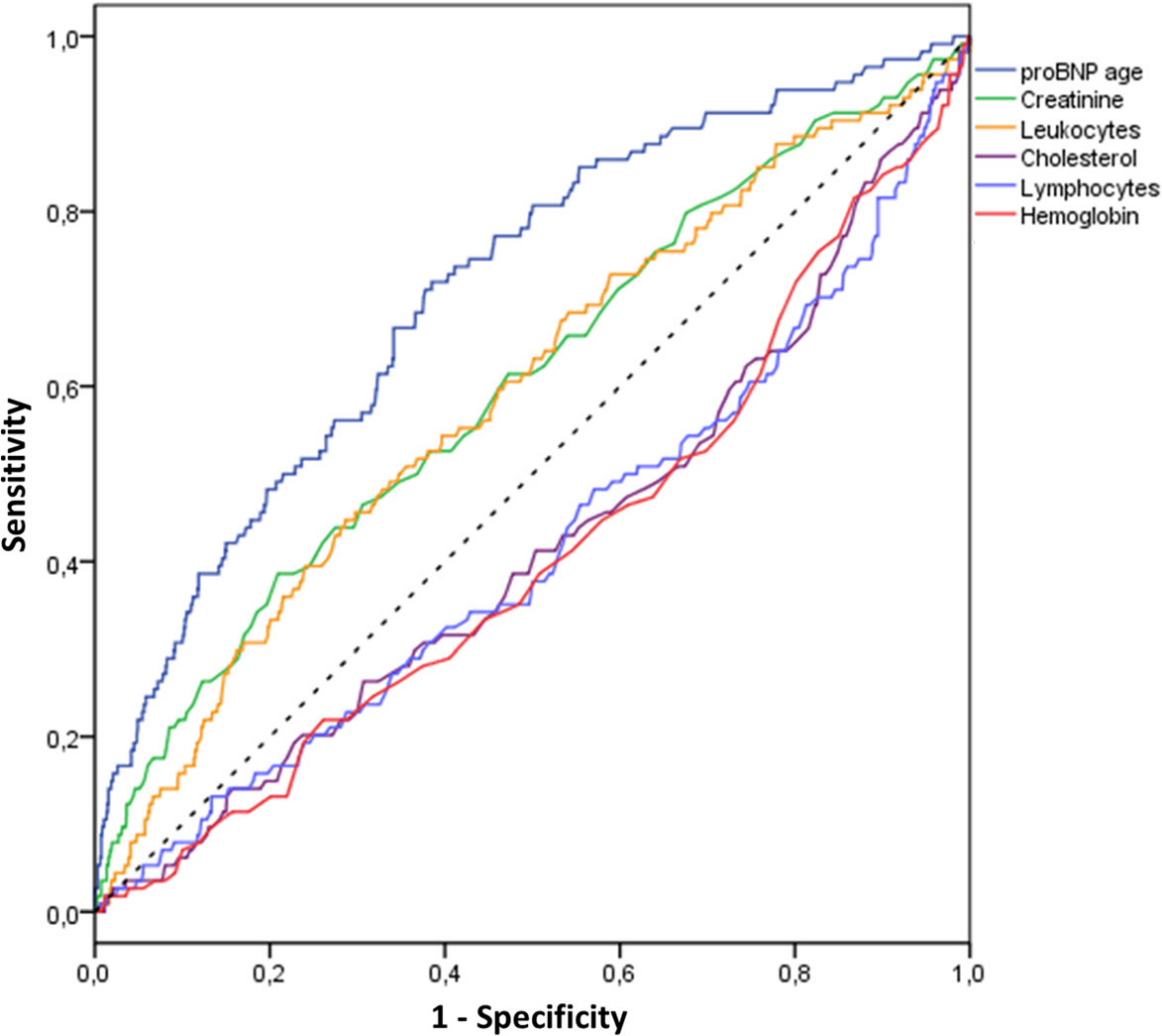
Receiver operating characteristic (ROC) curves of proBNP age (area under the curve (AUC) 0.712, 95% CI 0.661–0.762) and of the variables most associated with all-cause mortality: creatinine (AUC 0.603, 95% CI 0.546–0.660), leukocytes (AUC 0.592, 95% CI 0.535–0.649), cholesterol (AUC 0.419, 95% CI 0.361–0.476), lymphocytes (AUC 0.416, 95% CI 0.357–0.474), and hemoglobin (AUC 0.411, 95% CI 0.354–0.468)

Figure 3 shows the ROC curve of proBNP age in relation to all-cause mortality, together with the ROC curves of the other laboratory variables most associated with mortality (creatinine, leukocytes, cholesterol, lymphocytes and hemoglobin; see Table 3). The largest area was that under the ROC curve of proBNP age (0.712, 95% CI 0.661–0.762), confirming, with small increment, the AUC value of NT-proBNP that was calculated without considering sex (see Table 3).

**Fig. 4:**
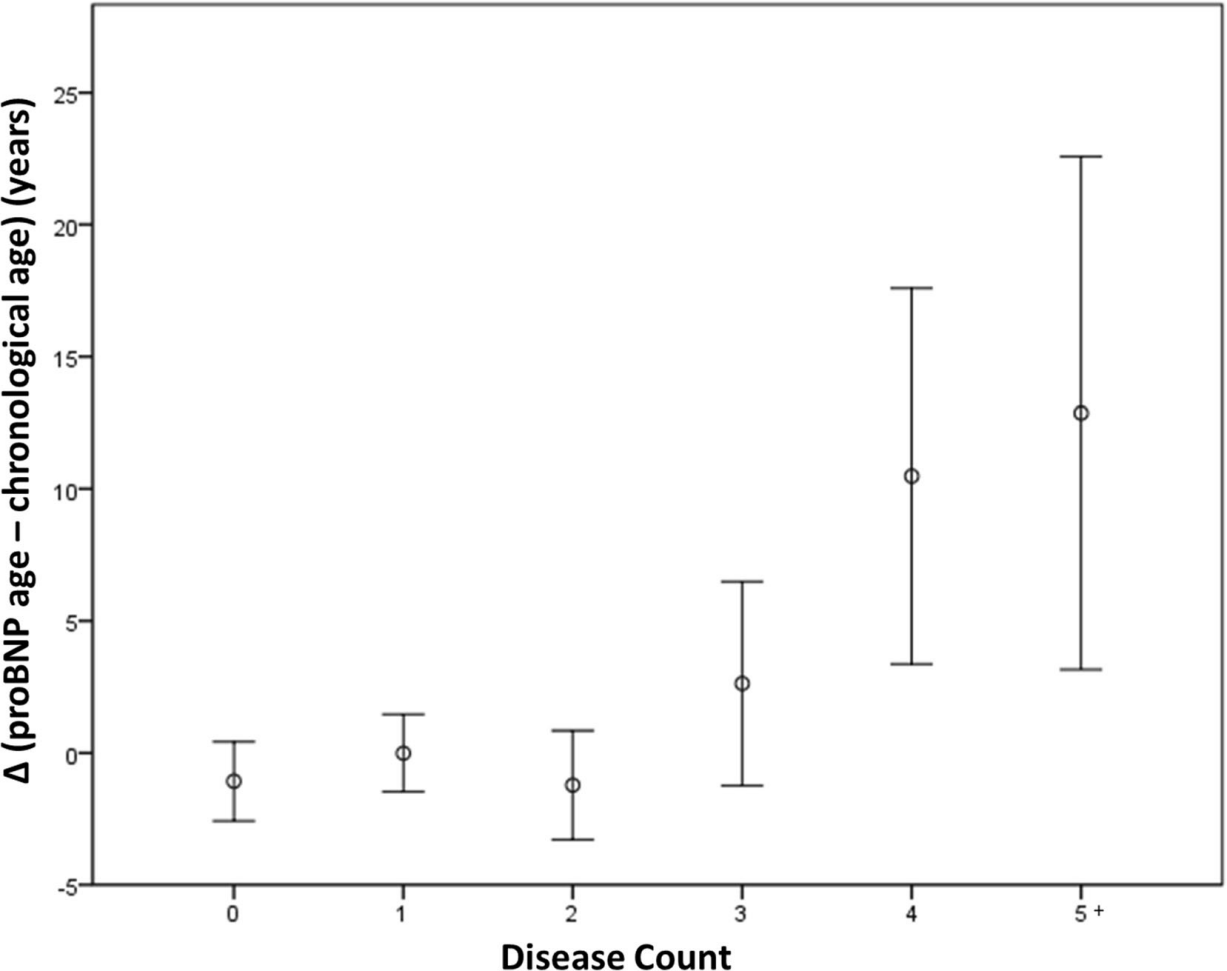
Relationship between Δ proBNP age–chronological age (mean values ± S.E.M.) and disease count

Finally, Table 5 shows percent mortality values according to proBNP age quintiles and quintiles of the difference (Δ) between pro-BNP age and chronological age. Hazard ratios with reference to the lowest quintile are also shown. Considering proBNP age absolute values, mortality increased significantly starting from the third quintile (proBNP age ≥ 69 years, mortality 7.9%, HR 2.6) and was maximum in the fifth quintile (proBNP age ≥ 85 years, mortality 22.8%, HR 7.9). Considering the values referred to chronological age (Δ), mortality increased significantly only for values of proBNP age that were 11 or more years greater than chronological age (mortality 20.5%, HR 3.5). In addition, Δ proBNP age–chronological age was also associated with morbidity: Fig. 4 shows a progressive rise of this parameter in the presence of 3 or more diseases (*P* ANOVA < 0.0001, *P* for linear trend = 0.0002).

**Table 5:** All-cause mortality according to proBNP age quintiles (absolute values or values referred to chronological age)

proBNP age quintile	Number	Deceased	Percent	HR (95% CI)	Δ proBNP age–chronological age quintile	Number	Deceased	Percent	HR (95% CI)
5 (≥ 85 years)	224	51	22.8	7.9 (3.6–17.5)	5 (≥ 11 years)	220	45	20.5	3.5 (1.9–6.4)
4 (≥ 75, < 85 years)	228	31	13.6	4.5 (2.0–10.2)	4 (≥ 3, < 11 years)	224	17	7.6	1.2 (0.6–2.4)
3 (≥ 69, < 75 years)	189	15	7.9	2.6 (1.0–6.3)	3 (≥ − 3, < 3 years)	198	22	11.1	1.8 (0.9–3.5)
2 (≥ 61, < 69 years)	219	10	4.6	1.4 (0.5–3.8)	2 (≥ − 11, < − 3 years)	216	16	7.4	1.2 (0.6–2.4)
1 (< 61 years)	219	7	3.2	1	1 (< − 11 years)	221	14	6.3	1

Hazard ratios are referred to first quintile

*CI* confidence interval, *HR* hazard ratio

## Discussion

In this study, among several biochemical health and disease indicators, NT-proBNP was the one most correlated with chronological age and all-cause mortality (in the case of mortality, with even better results than chronological age and disease count). Thus, using simple formulas separately for the two sexes, starting from NT-proBNP values, we obtained an estimate of biological age, proBNP age, which, unlike NT-proBNP, can be considered equally valid for men and women.

ProBNP age is probably not an accurate measure of real biological age, but it may equally serve as surrogate end point in epidemiological studies and even for personal use: (1) it is easily interpretable, (2) it may be assessed longitudinally to study its course (spontaneous, or possibly influenced by treatment), and (3) it is a measure of the risk of morbidity and all-cause death. In particular, anyone can calculate his/her proBNP age, according to his/her NT-proBNP value and sex, using the coefficients provided in the Results (formulas (3) and (4)). Of course, having a proBNP age equal or younger than chronological age will be preferable, although significantly increased mortality was only associated with an excess of 11 or more years with respect to chronological age. In absolute, the lowest risk of death was associated with a proBNP age < 69 years, while the highest risk was associated with a proBNP age ≥ 85 years.

### Non-cardiovascular determinants of NT-proBNP and association with non-cardiovascular mortality

NT-proBNP, like BNP, is released from cardiomyocytes undergoing wall stress or ischemia and, therefore, it is a known indicator of heart failure, atrial fibrillation, acute coronary syndromes, and cardiovascular mortality (McDonagh et al. 2004; Galvani et al. 2004; Ponikowski et al. 2016; Wang et al. 2004; Ulimoen et al. 2009). Other cardiovascular factors directly associated with an increase in these natriuretic peptides are systemic and pulmonary arterial hypertension, myocardial hypertrophy, pulmonary embolism, myocarditis, valve and congenital heart diseases, tachyarrhythmias, electric cardioversion, and beta-blocker treatment (Ponikowski et al. 2016), while heart rate is inversely correlated (Loke et al. 2003). However, several studies have shown that these natriuretic peptides are also associated with non-cardiovascular mortality (Muscari et al. 2013) and all-cause mortality in subjects free of cardiovascular diseases (McKie et al. 2006; Wannamethee et al. 2011; Muscari et al. 2013), and the same occurred in this study, from which the subjects with a history of heart failure and atrial fibrillation had been excluded. In fact, also many extra-cardiac determinants are associated with an increment of these natriuretic peptides, such as renal insufficiency, anemia, diabetes, ischemic stroke, subarachnoid hemorrhage, paraneoplastic syndromes, chronic obstructive pulmonary disease (COPD), sepsis, burns, severe hormonal dysfunctions, and liver diseases like cirrhosis (Ponikowski et al. 2016), while obesity, especially visceral, and hepatic steatosis are associated with lower serum levels (Luchner et al. 2013; Muscari et al. 2006). The association between NT-proBNP levels and non-cardiovascular mortality might also have simpler explanations, in addition to the above non-cardiac determinants, such as the concomitance of inapparent myocardial impairment, or coding errors in death certificates.

### Association between age and NT-proBNP

The levels of NT-proBNP progressively increase with age even in healthy subjects (Redfield et al. 2002; Wang et al. 2002), as was also confirmed by a long-term longitudinal study (Luchner et al. 2013). The relationship between age and NT-proBNP is of exponential type (Redfield et al. 2002; Luchner et al. 2013) (it becomes linear after logarithmic transformation of values), like the relationship between age and mortality (at least before the age of 105 years) (Barbi et al. 2018). The European guidelines for heart failure suggest a unique cutoff of NT-proBNP, independent of age, below which the probability of heart failure is considered neglectable: 125 pg/ml for non-acute heart failure and 300 pg/ml for acute heart failure (Ponikowski et al. 2016). Nevertheless, in our sample, the first cutoff was exceeded, on average, at the age of 69 by women and of 73 by men, and the second cutoff was exceeded at the age of 87 by women and of 83 by men, in the absence of heart failure. Indeed, several authors have suggested to adopt different cutoffs for the diagnosis of heart failure, taking into account the progressive increase of natriuretic peptides with age (Nageh et al. 2002; Hildebrandt et al. 2010; Keyzer et al. 2014). The reason for this age-dependent increment in both sexes is unknown. It might be related to aging-associated processes, such as myocardial hypertrophy and fibrosis (Boyle et al. 2011), or the reduction of clearing mechanisms of natriuretic peptides from plasma (Clark et al. 1991).

### Association between female sex and NT-proBNP

The association of higher levels of NT-proBNP with female sex is well known (Redfield et al. 2002; Loke et al. 2003), so that some laboratories have proposed differentiated normal values for the two sexes. In our sample, the regression line between age and logarithm of NT-proBNP in women had an intercept greater than that of men, so that, for the major part of life, for the same age, women had higher NT-proBNP levels and, for the same NT-proBNP level, they were younger than men. However, the regression line in men was steeper than that in women, so after the age of 78.3, the values in men exceeded those in women. To our knowledge, this is the first time that the overtaking of NT-proBNP values in women by values in men is documented, although Raymond et al. in 2003 reported a significant difference, in favor of women, only up to 70 years of age (Raymond et al. 2003) and, more recently, in a sample with a mean age lower than that of our sample, higher values in women were documented up to 50 years of age (Hamada et al. 2017). In general, the causes of the different NT-proBNP levels in the two sexes are poorly understood. In a previous study, we found that NT-proBNP levels are inversely related to hematocrit, which is lower in women than in men (Muscari et al. 2006). Other possible explanations concern the lower levels of plasma renin in women (Kuroski de Bold 1999) or the effects that female sex hormones may have on gene expression of natriuretic peptides (Hong et al. 1992).

### Is it possible to modify blood levels of natriuretic peptides with anti-aging interventions?

In patients with heart failure and high natriuretic peptide levels, it is certainly possible to obtain a decrement of such levels with heart failure treatment (Richards and Troughton 2004). However, in subjects without heart disease, it is unknown whether reducing these peptide levels, or at least slowing their progression, is possible with anti-aging treatments, also, and mainly because at present such treatments are only hypothetical. The only anti-aging treatment of documented efficacy is caloric restriction with adequate nutrition (Dorling et al. 2020). In rat, regimens of dietetic restriction prevented the age-related increase of ANP stores in atrial tissue and lowered plasma ANP levels (Cavallini et al. 1995). To our knowledge, there are no equivalent studies in man concerning BNP or NT-proBNP.

### Other findings

As far as the other laboratory parameters considered are concerned, creatinine, after NT-proBNP, was the variable most associated with mortality, in addition of being significantly correlated with age, as already reported by others (Liu et al. 2018). In addition, the inverse association of hemoglobin, lymphocytes, cholesterol, and albumin with both age and mortality was also confirmed, probably because in elderly people, low values of these variables are markers of frailty. However, in a multivariate analysis including NT-proBNP, none of these laboratory variables was independently associated with mortality.

### Previous studies on laboratory or clinical variables and biological age

The attempts to obtain an indicator of biological age starting from laboratory or clinical, usually multiple, variables, have been numerous. Sometimes indices exclusively composed by clinical-anamnestic data, without any laboratory variable, have been proposed, such as the deficit index (FI34) by Kim and Jazwinski (2015). This index was based on 34 variables, such as anemia history, cataract history, asthma history, COPD history, kidney disease history, BMI, Mini Mental State Examination, Activities of Daily Living, Geriatric Depression Scale, and self-assessment of health status. This index was well associated with mortality and disease count, which is understandable considering that it includes clinical-anamnestic references to all main human pathologies. It is certainly an accurate assessment of health, frailty, and comorbidity status, which however is more associated with the effects of diseases rather than with the aging process. In addition, considering the prevalence of anamnestic parameters (24 out of 34), it might be poorly influenced by possible treatments. A similar and even more complex index (40 items) of multidimensional frailty has been proposed for the Italian elderly population (Abete et al. 2017). This index also includes Instrumental Activities of Daily Living, weight loss, peak expiratory flow, Mini Nutritional Assessment, and an accurate evaluation of walking ability and muscle strength, with good associations with mortality, disability, and hospitalization rate.

On the other hand, other studies utilized common laboratory parameters, such as creatinine, CRP, albumin, cholesterol, and leukocytes, which were combined by mathematical functions to produce biological age directly or indirectly through intermediate calculation of mortality (Yoo et al. 2017; Belsky et al. 2017; Liu et al. 2018; Waziry et al. 2019). Sometimes, systolic blood pressure was included in the index (Belsky et al. 2017; Waziry et al. 2019), although, considering the spontaneous variability of this parameter, random variations of biological age could be generated. In addition, systolic blood pressure increases with age, but its relationship with mortality, in the elderly and in some pathologies, may be of inverse type (Muscari et al. 2013; Weiss et al. 2010; Okin et al. 2012). In some cases, among the predictive parameters of biological age, also chronological age was included, thus obtaining very good correlations between chronological age and the index of biological age, which might be, however, a biased procedure (Liu et al. 2018). These indices have at least the potentiality to improve with some form of anti-aging treatment, and, in fact, after 2 years of caloric restriction, in the CALERIE trial, Belsky et al. (2017) obtained an improvement of two indices of biological age that were mainly based on biochemical variables. However, cholesterol, uric acid, blood glucose, and systolic blood pressure may also be influenced by drugs, which may cause relevant fluctuations in the calculation of biological age according to Belsky’s formulas. In addition, very reliable and validated multiparametric risk profiles have been available for many years (D’Agostino et al. 2008), and the transformation of a multiparametric mortality risk into years of biological age might appear a useless procedure. Finally, none of these attempts to estimate biological age included BNP or NT-proBNP, which carry a great part of the information associated with other biochemical parameters. In fact, we have documented that the association with mortality of many of these parameters disappeared when the multivariate analysis included NT-proBNP, thus making useless the creation of a complicate multiparametric index.

### Limitations

First of all, this study needs a confirmation, being the first study that has proposed NT-proBNP as a possible indicator of biological age. Thus, it would be advisable to seek to reproduce our assessments in a different and possibly larger population. In addition, the associations of pro-BNP age with disease-associated mortalities and specific pathologies should be verified. It would also be desirable a direct comparison with other modalities of biological age assessment. Probably, proBNP age cannot reliably be calculated in subjects with very high NT-proBNP levels, as is the case in the presence of heart failure or atrial fibrillation. There is also the need to extend the assessment to younger subjects (the relationships found in people over 65 years of age cannot automatically be extrapolated downwards). Finally, we found that NT-proBNP is probably the single best predictor of mortality and that it is also associated with disease count, but whether it truly reflects some of the biological mechanisms of aging is uncertain and its relationship with other age-related outcomes, such as reduced walking speed, physical disability, and cognitive impairment, should be assessed in further studies.

## Conclusions

Among all the laboratory variables considered in this study, NT-proBNP was the one most associated with chronological age and all-cause mortality. Thus, through simple formulas, we have proposed a method that allows the transformation of NT-proBNP, which varies differently in the two sexes, into a value of “pro-BNP age,” which is independent of sex and easily interpretable both as absolute value and in relation to chronological age. If confirmed by other studies, this indicator of biological age, obtained by a simple blood measurement, might prove useful as a surrogate end point in the assessment of anti-aging treatments.

## Data Availability

The data that support the findings of this study are available from the corresponding author upon reasonable request.
